# Efficacy of fosinopril and amlodipine in pediatric primary hypertension: a single-center observational study

**DOI:** 10.3389/fped.2023.1247192

**Published:** 2023-10-27

**Authors:** Hui Wang, Lin Shi, Yao Lin, Yuting Wang, Wenquan Niu, Yaqi Li

**Affiliations:** ^1^ Children's Hospital Capital Institute of Pediatrics, Chinese Academy of Medical Sciences & Peking Union Medical College, Beijing, China; ^2^ Department of Cardiology, Children’s Hospital Capital Institute of Pediatrics, Beijing, China; ^3^ Center for Evidence-Based Medicine, Capital Institute of Pediatrics, Beijing, China

**Keywords:** fosinopril, amlodipine, efficacy, primary hypertension, children

## Abstract

**Objective:**

Fosinopril and amlodipine are commonly prescribed as first-line pharmacotherapeutic agents for pediatric hypertension, but there is a lack of comparative studies regarding the efficacy of these two drugs. We aimed to evaluate and compare the efficacy of fosinopril and amlodipine monotherapy in pediatric primary hypertension.

**Methods:**

This was a single-center, bidirectional observational study. A total of 175 children and adolescents with primary hypertension receiving antihypertensive monotherapy from July 2020 to February 2023 were enrolled. According to antihypertensive drugs, they were divided into the fosinopril group (*n* = 96) and the amlodipine group (*n* = 79). Subgroup analysis was performed to compare the efficacy of the two groups in terms of blood pressure (BP) control rates and reductions following a 4-week treatment.

**Results:**

After 4 weeks of treatment, both groups achieved significant reductions in systolic BP (SBP) and diastolic BP (DBP) by more than 18 mmHg and 6 mmHg, respectively, with BP control rates of 61.5% in the fosinopril group and 59.5% in the amlodipine group, revealing no significant differences in the antihypertensive efficacy between the two groups except for DBP control rate (FDR adjusted *P *> 0.05). Further subsequent subgroup analyses revealed that the reductions in SBP and DBP in the fosinopril group were significantly greater than those in the amlodipine group in patients of females and hypo-HDL-cholesterolemia (FDR adjusted *P* < 0.05), and there was a trend of difference, although not significant, in patients with central obesity and insulin resistance (IR) (FDR adjusted 0.05 < *P* ≤ 0.1). However, there were no significant differences in treatment efficacy in patients without these characteristics. Furthermore, hypertriglyceridemia did not exhibit a significant association with the difference in treatment efficacy between the two medications (FDR adjusted *P* > 0.05).

**Conclusions:**

Fosinopril and amlodipine monotherapy were both effective in pediatric primary hypertension during a short-term follow-up. Fosinopril may be particularly effective in reducing BP in hypertensive patients of females, central obesity, IR, and hypo-HDL-cholesterolemia. These findings indicate that optimizing antihypertensive medication selection based on the individualized characteristics of children with hypertension may improve the efficacy of antihypertensive treatment.

## 1. Introduction

Increasing evidence showed that hypertension is becoming more common in children and adolescents, which may be related to the increased incidence of obesity (1, 2), and also due to the widespread use of blood pressure (BP) measurement (3). Pediatric hypertension follows a certain trajectory that increases the risk of cardiovascular disease and kidney disease in adulthood (4, 5). Therefore, timely diagnosis and effective treatment of hypertension in children are important.

Despite the increasing need for antihypertensive medication among children with primary hypertension (6), and the number of antihypertensive agents on the market has increased significantly (7), current studies on antihypertensive drugs are mostly focused on adults, and there is limited knowledge about antihypertensive drug choices in children and adolescents. Guidelines and scientific statements generally recommend Angiotensin-Converting Enzyme Inhibitors (ACEI), Calcium Channel Blockers (CCB), Angiotensin Receptor Blockers (ARB), and thiazide diuretics as initial treatment for hypertension in children and adolescents (8–10). However, the comparison of the efficacy of various antihypertensive drugs in children has not been fully documented by large-scale clinical study, and pediatricians usually choose drugs based on the experience of clinical practice in adults. In fact, the pharmacokinetics of children and adults are different, which means that the antihypertensive medication regimens for children cannot be completely referenced to that for adults.

Fosinopril is a long-acting agent of ACEI on the Food and Drug Administration (FDA) Pediatric Priority List (11), and is recommended for use in hypertensive children by the 2018 Chinese Guidelines on Hypertension Prevention and Treatment (12). Amlodipine is the only CCB antihypertensive drug recommended by FDA for hypertensive children (13), which has a long-lasting effect and is well tolerated. As the first-line treatment for hypertension in children, there is a lack of comparative studies of the two drugs. Thus, children and adolescents diagnosed with primary hypertension and treated with fosinopril or amlodipine monotherapy were enrolled in this study. BP levels were compared after 4 weeks in order to provide a reference for the selection of appropriate antihypertensive drugs in children with primary hypertension.

## 2. Methods

### 2.1. Study population

This is an ongoing single-center, bidirectional cohort study conducted at Capital Institute of Pediatrics. A total of 175 children and adolescents with primary hypertension receiving antihypertensive monotherapy from July 2020 to February 2023 were included in the final analysis population. Antihypertensive drug therapy is indicated for patients with at least one of the following: (a) symptomatic hypertension, (b) hypertensive target organ damage, (c) hypertension stage 2, or (d) hypertension stage 1 with inadequate response to 6-month lifestyle modification. The study population was divided into two groups: the fosinopril group (*n* = 96) and the amlodipine group (*n* = 79), with no specific drug intentionally prescribed for the purpose of this comparative study. All subjects completed necessary tests, as described in our previous study (14). Patients with secondary hypertension caused by renal disease, endocrine disease, central nervous system disease or medication, and patients with primary hypertension receiving nonpharmacologic treatment or other drugs treatment were excluded.

### 2.2. BP measurement and diagnostic criteria

BP was measured with mercury sphygmomanometer in all subjects and graded according to the 2018 Chinese Guidelines for Prevention and Treatment of Hypertension (12), and 24-h ambulatory blood pressure monitoring was used to exclude white coat hypertension (10, 15). Hypertension is diagnosed when systolic blood pressure (SBP) and/or diastolic blood pressure (DBP) ≥95th percentile for age, sex, and height at least 3 separate occasions. Further, the stages are classified as stage 1 when blood pressure is ≥95th but <99th percentile + 5 mmHg for age, sex, and height, and stage 2 when SBP or DBP ≥99th percentile + 5 mmHg for age, sex, and height.

### 2.3. Clinical data collection

All data was collected from the electronic medical record system. General data included age, sex, family history, height, weight, waist circumference, and calculated body mass index (BMI). Obesity was diagnosed when BMI exceeded the 95th BMI in children of the same age and sex, and when waist circumference/height >0.48 in males or 0.46 in females, central obesity was determined (16). The laboratory parameters such as fasting blood glucose (FBG), fasting insulin, uric acid, creatinine, triglycerides, total cholesterol, high-density lipoprotein cholesterol (HDL-C), and low-density lipoprotein cholesterol were recorded. Echocardiographic data were recorded and left ventricular mass index and relative wall thickness were calculated as described in our previous study (14). Urine microalbumin and urine creatinine were recorded, and albumin/creatinine quotient and estimated glomerular filtration rate (eGFR) were calculated as described elsewhere (17).

### 2.4. Treatment protocol and follow-up

**Figure 1:**
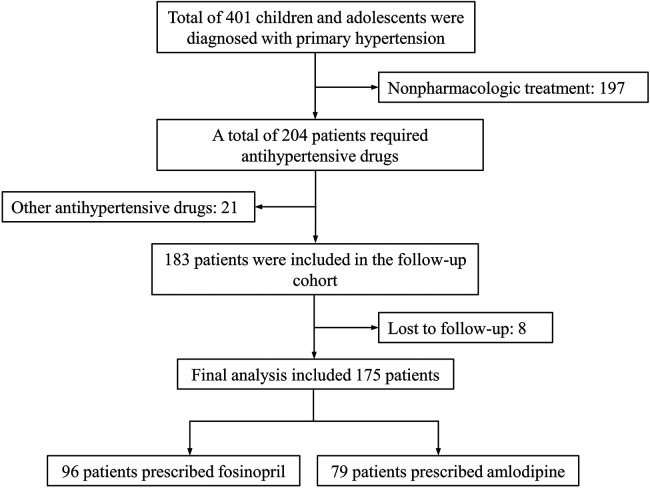
The study flowchart.

An initial dosage of 10 mg daily fosinopril or 5 mg daily amlodipine was prescribed to all subjects. BP was evaluated weekly, and if necessary, the dosage was adjusted from the second week of treatment until the goal BP (95th percentile for age, sex, and height) was achieved or the maximum dose (40 mg daily for fosinopril and 10 mg daily for amlodipine) was reached. According to previous studies and our clinical observations, 4-week treatment of fosinopril or amlodipine can lead to a stable BP level (18, 19). For those patients who have reached the maximum dosage but not achieved the goal BP, alternative or supplementary other antihypertensive agents should be considered after 4 weeks of treatment. Hence, the last follow-up with BP measured was completed after 4-week monotherapy. We calculated BP control rate as the ratio of the number of subjects achieved goal BP to the total subjects in each group. The study flowchart is shown in Figure 1.

### 2.5. Subgroup analyses

Subgroup analyses were performed on efficacy parameters (BP control rate and BP reduction) to compare the differences between fosinopril and amlodipine for subgroups of males and females, as well as subgroups of patients with central obesity and those without. Furthermore, homeostasis model assessments of insulin resistance (HOMA-IR) score was calculated following the formula (HOMA-IR = fasting insulin (μU/ml) × FBG (mmol/L)/22.5), and subjects were classified into subgroups of IR (>3.16) and non-IR (≤3.16) in accordance with this score (20). Additionally, subjects were divided into subgroups of hypertriglyceridemia (yes/no), hypo-HDL-cholesterolemia (yes/no) according to 2022 expert consensus on diagnosis and management of dyslipidemia in children (21).

### 2.6. Statistical analyses

The Shapiro–Wilk test was utilized to assess the normality of the data. Mean ± standard deviation (SD) was used to express parametric continuous data, and independent t test and paired t test were employed for between- and within-group comparisons, respectively. Non-parametric data was presented as median (the interquartile range) and analyzed using the Mann–Whitney test. Categorical variables were compared using the Chi-squared test and Fisher exact test. *P*-values for treatment efficacy were adjusted by false discovery rate (FDR), which was calculated according to Benjamini-Hochberg, and all *P* < 0.05 were considered statistically significant. All statistical analyses were done using SPSS 25.0 and GraphPad Prism 8 software.

## 3. Results

A total of 175 subjects were included in this study, including 139 males and 36 females, with age ranged 13.0 (2.0) years, of whom 136 (77.7%) were central obesity. The median initial dosages of fosinopril and amlodipine were 0.27 and 0.06 mg/kg, respectively. The baseline characteristics of the study participants are shown in Table 1, and there were no statistically significant differences in those between the fosinopril and amlodipine groups.

**Table 1:** Baseline characteristics of the study participants.

Characteristic	Fosinopril (*n* = 96)	Amlodipine (*n* = 79)	*P*-value
Male (*n*, %)	74 (77.1)	65 (82.3)	0.398
Age (years)	13.0 (2.0)	13.0 (3.0)	0.902
Family history (*n*, %)	53 (55.2)	45 (57.0)	0.816
Baseline height (m)	1.71 (0.11)	1.72 (0.16)	0.636
Baseline weight (kg)	82.81 ± 17.69	84.05 ± 21.11	0.672
Baseline BMI (kg/m^2^)	28.10 ± 4.59	28.65 ± 5.16	0.455
Obesity (*n*, %)	79 (82.3)	62 (78.5)	0.526
Central obesity (*n*, %)	75 (78.1)	61 (77.2)	0.886
Baseline SBP (mmHg)	144.5 (13.5)	143.0 (13.0)	0.181
Baseline DBP (mmHg)	83.0 (17.5)	80.0 (12.0)	0.117
ISH (*n*, %)	33 (34.4)	34 (43.0)	0.241
Stage 2 hypertension (*n*, %)	87 (90.6)	76 (96.2)	0.146
Fasting blood glucose (mmol/L)	4.47 (0.63)	4.62 (0.56)	0.098
Fasting insulin (μU/ml)	21.30 (16.00)	22.40 (14.10)	0.834
HOMA-IR	4.29 (3.04)	4.41 (3.16)	0.930
Serum creatinine (µmol/L)	53.41 ± 10.59	54.62 ± 13.10	0.510
Serum uric acid (µmol/L)	453.5 ± 108.5	454.9 ± 88.1	0.930
Total cholesterol (mmol/L)	3.91 ± 0.71	4.06 ± 0.81	0.196
Triglyceride (mmol/L	1.19 (0.60)	1.30 (0.84)	0.207
HDL-C (mmol/L)	1.03 (0.28)	1.04 (0.30)	0.857
LDL-C (mmol/L)	2.45 (0.91)	2.68 (1.06)	0.125
ACR (mg/g)	4.94 (6.68)	5.41 (5.01)	0.627
eGFR (ml/min/1.73 m^2^)	105.5 (24.7)	107.6 (26.2)	0.816
LVMI (g/m^2.7^)	29.01 (9.26)	28.16 (8.73)	0.713
RWT	0.33 (0.05)	0.31 (0.05)	0.706

BMI, body mass index; SBP, systolic blood pressure; DBP, diastolic blood pressure; ISH, isolated systolic hypertension; HOMA-IR, homeostasis model assessments of insulin resistance; HDL-C, high-density lipoprotein cholesterol; LDL-C, low-density lipoprotein cholesterol; ACR, urinary albumin/creatinine quotient; eGFR, estimated glomerular filtration rate; LVMI, left ventricular mass index; RWT, relative left ventricular wall thickness. The continuous variables were presented as mean ± standard deviation or median (interquartile range).

**Figure 2:**
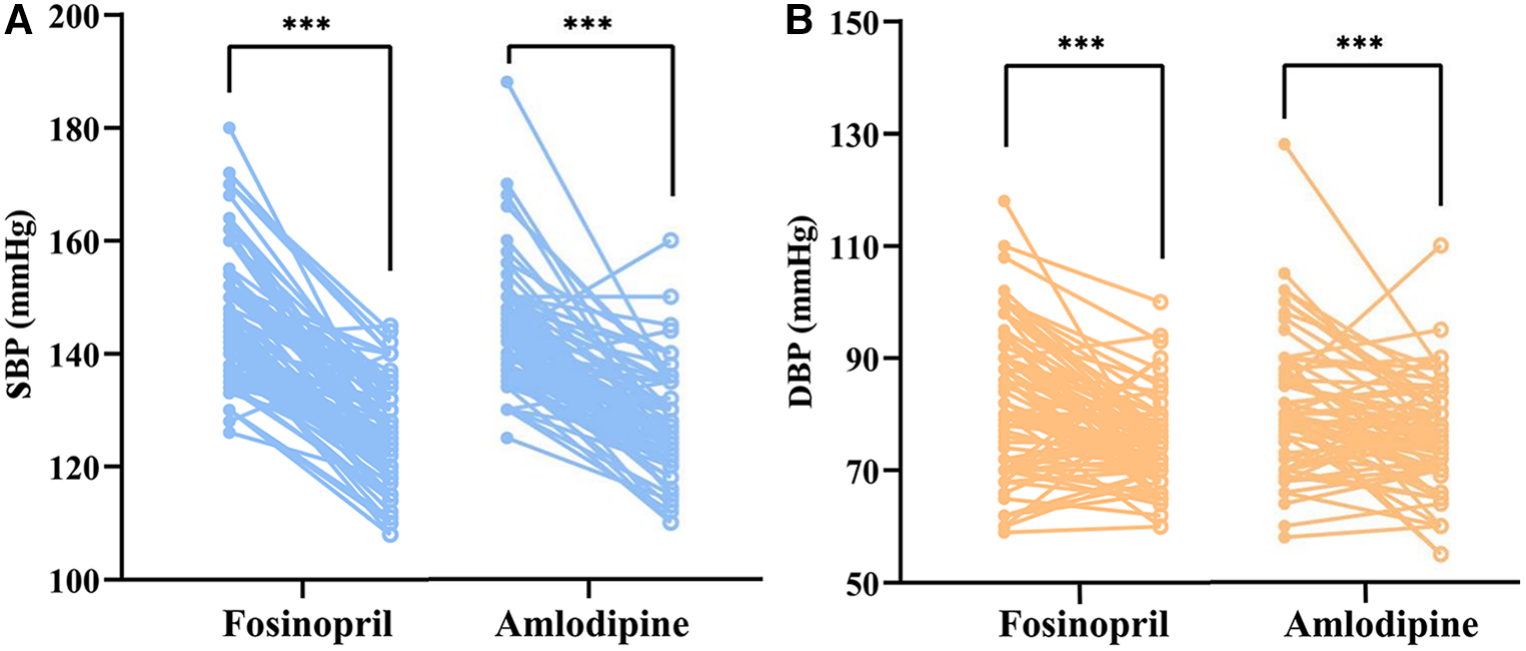
Paired dot plots compared changes in SBP (left) and DBP (right) before and after treatment with fosinopril and amlodipine. SBP, systolic blood pressure; DBP, diastolic blood pressure. *** indicates *p *< 0.001, compared with baseline.

After 4 weeks of treatment, both the two groups achieved significant SBP and DBP reductions (*P* < 0.001) (Figure 2), with BP control rates of 61.5% in the fosinopril group and 59.5% in the amlodipine group. Furthermore, SBP decreased by 21.1 mmHg and 18.0 mmHg in the fosinopril and amlodipine groups, and DBP decreased by 9.1 mmHg and 6.1 mmHg, respectively, leading to a significant 10% difference in the extent of SBP and DBP reductions between the two groups (*P* = 0.083 and 0.093), but no differences in BP and SBP control rates, except a higher DBP control rate in the fosinopril group (Table 2). After adjustment, there was no significant difference in treatment efficacy between the two groups (FDR adjusted *P *> 0.05). At week 4, the median dosage of fosinopril was increased in 53 patients to 0.35 mg/kg, while increased in 30 patients to 0.09 mg/kg of amlodipine. Furthermore, considering the potential effect of fosinopril on renal function, we followed up 82 patients prescribed fosinopril and found no significant difference in eGFR levels between before and after treatment [108.3 (24.8) vs. 109.5 (27.7) ml/min/1.73 m^2^, *P* = 0.314].

**Table 2:** Comparison of BP changes and control rates between the fosinopril and amlodipine group.

Characteristic	Fosinopril (*n* = 96)	Amlodipine (*n* = 79)	*P*-value	Adjusted *P*-value
Posttreatment SBP (mmHg)	125.0 (12.0)^*^	125.0 (10.0)^*^	0.710	
Posttreatment DBP (mmHg)	75.0 (8.0)^*^	75.0 (10.0)^*^	0.339	
d-SBP (mmHg)	21.1 ± 10.91	18.0 ± 12.11	0.083	0.155
d-DBP (mmHg)	9.1 ± 12.17	6.1 ± 11.19	0.093	0.155
BP control rate (*n*, %)	59 (61.5)	47 (59.5)	0.791	0.973
SBP control rate (*n*, %)	61 (63.5)	50 (63.3)	0.973	0.973
DBP control rate (*n*, %)^a^	49 (77.8)	25 (55.6)	0.014	0.07

BP, blood pressure; SBP, systolic blood pressure; DBP, diastolic blood pressure; d-SBP, decrease of SBP from baseline to week 4; d-DBP, decrease of DBP from baseline to week 4. The continuous variables were presented as mean ± standard deviation or median (interquartile range).

^a^ Indicates DBP control rate in systolic and diastolic hypertension.

^*^ Indicates *p *< 0.001, compared with baseline.

**Figure 3:**
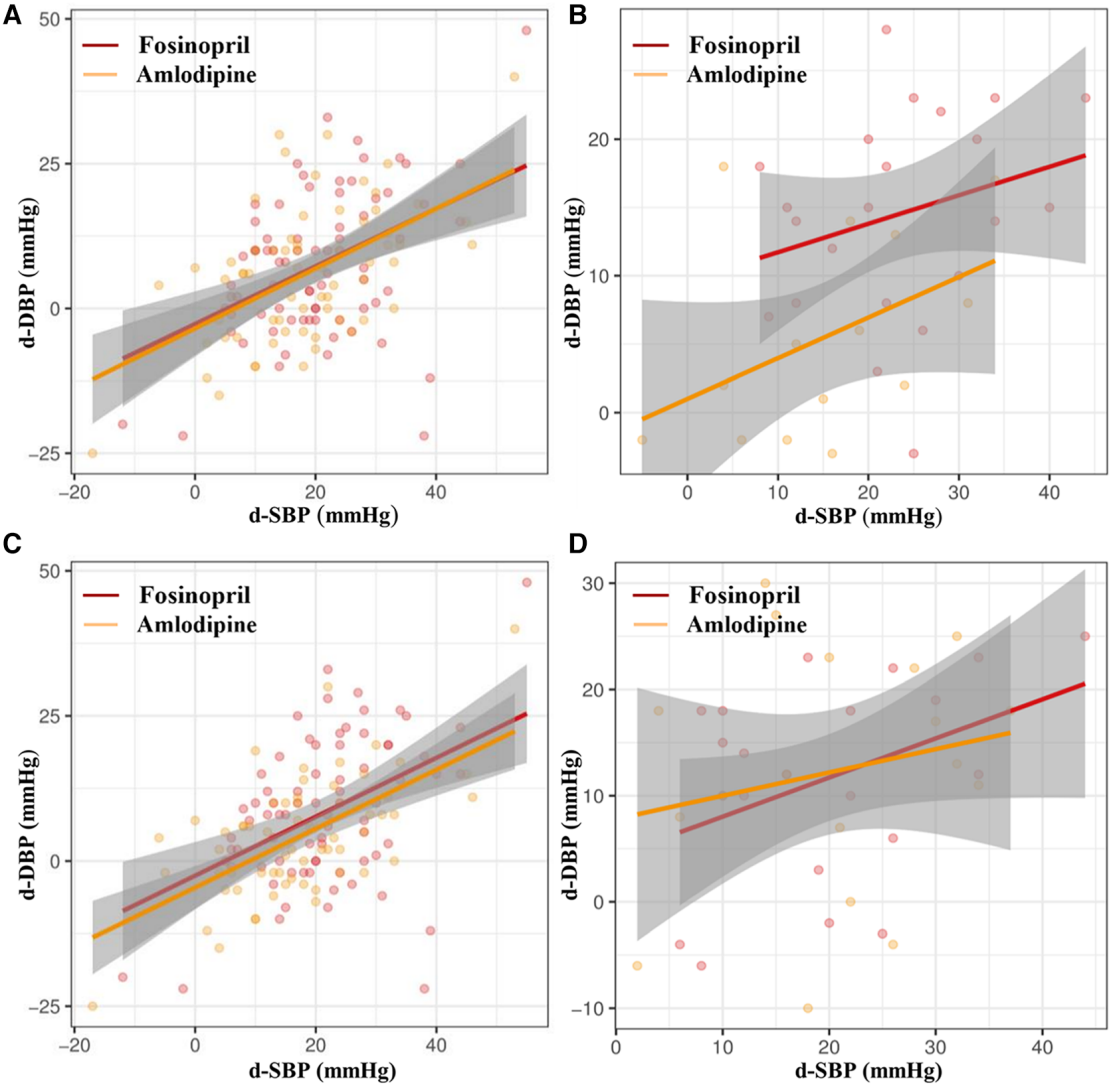
Scatter diagrams of correlation analysis of d-SBP and d-DBP of fosinopril and amlodipine in (**A**) male and (**B**) female subgroups, and (**C**) central obesity and (**D**) non-central obesity subgroups. d-SBP, decrease of SBP from baseline to week 4; d-DBP, decrease of DBP from baseline to week 4.

Subgroup analysis showed that the fosinopril group had greater extent of BP reduction and higher BP and DBP control rates than the amlodipine group in female patients (FDR adjusted *P* < 0.05), with a borderline difference in SBP control rate (FDR adjusted *P* = 0.056), but these differences were not statistically significant in male patients. Additionally, in patients with central obesity, fosinopril group had a tendency to a greater reduction in SBP and DBP (FDR adjusted *P* = 0.07) (Table 3). To further illustrate these findings, a scatterplot comparing the decreases in SBP and DBP distributions between the fosinopril and amlodipine groups in gender and central obesity subgroups is presented in Figure 3. The fitted regression lines showed good discrimination in the subgroups of females and central obesity, suggesting that there are significant differences in the degree of SBP and DBP reductions between the fosinopril and amlodipine groups.

**Table 3:** Subgroup analyses for BP reductions (mmHg) and control rates (%) after treatment with fosinopril or amlodipine.

Subgroup	Characteristic	Fosinopril	Amlodipine	*P*-value	Adjusted *P*-value
Gender					
Male (*n* = 139)	d-SBP	20.4 ± 11.19	18.6 ± 12.35	0.383	0.693
	d-DBP	7.5 ± 12.82	6.2 ± 11.89	0.546	0.546
	BP control rate	44 (59.5)	43 (66.2)	0.416	0.693
	SBP control rate	46 (62.2)	45 (69.2)	0.382	0.693
	DBP control rate^a^	34 (75.6)	23 (67.6)	0.437	0.546
Female (*n* = 36)	d-SBP	23.3 ± 9.81	15.1 ± 10.87	0.025	0.031
	d-DBP	14.5 ± 7.62	5.5 ± 7.39	0.001	0.003
	BP control rate	15 (68.2)	4 (28.6)	0.020	0.031
	SBP control rate	15 (68.2)	5 (35.7)	0.056	0.056
	DBP control rate^a^	15 (83.3)	2 (18.2)	0.001	0.003
Central obesity					
Yes (*n* = 136)	d-SBP	21.5 ± 11.12	17.3 ± 12.50	0.043	0.073
	d-DBP	8.4 ± 12.78	4.2 ± 10.47	0.044	0.073
	BP control rate	45 (60.0)	33 (54.1)	0.489	0.531
	SBP control rate	47 (62.7)	35 (57.4)	0.531	0.532
	DBP control rate^a^	37 (78.7)	16 (50.0)	0.008	0.040
No (*n* = 39)	d-SBP	19.6 ± 10.25	20.4 ± 10.66	0.820	1.000
	d-DBP	11.6 ± 9.51	12.3 ± 11.61	0.836	1.000
	BP control rate	14 (66.7)	14 (77.8)	0.442	1.000
	SBP control rate	14 (66.7)	15 (83.3)	0.412	1.000
	DBP control rate^a^	12 (75.0)	9 (69.2)	1.000	1.000

BP, blood pressure; SBP, systolic blood pressure; DBP, diastolic blood pressure; d-SBP, decrease of SBP from baseline to week 4; d-DBP, decrease of DBP from baseline to week 4.

^a^ Indicates DBP control rate in systolic and diastolic hypertension. The continuous variables were presented as mean ± standard deviation.

Furthermore, we observed that SBP and DBP reductions in the fosinopril group were significantly greater than those of the amlodipine group in patients with hypo-HDL-cholesterolemia (FDR adjusted *P* < 0.05). In patients with IR, the control rate of DBP was significantly higher in the fosinopril group than in the amlodipine group, and there was a trend, although not significant, difference in the reduction of SBP and DBP between the two groups (FDR adjusted *P* = 0.080 and 0.100). However, no significant differences on treatment efficacy were found in patients without these characteristics (FDR adjusted *P* > 0.05). In addition, hypertriglyceridemia did not appear to show a relationship with the difference in treatment efficacy between the two medications (FDR adjusted *P* > 0.05). Notably, there were no significant differences in baseline characteristics between the treatment arms in all subgroups (*P* > 0.05), except for the gender in patients without central obesity. All analyses are presented in Supplementary Table S1.

It is worth mentioning that there were no occurrences of hypotensive events or serious adverse events occurred throughout the period of treatment. 2 patients (2.4%) in the fosinopril group suffered transient dry cough with dosage of 20 mg once daily, and 1 patient (1.3%) from the amlodipine group complained of palpitation after receiving the initial dose of amlodipine with no abnormality detected on the electrocardiogram. The incidence of adverse events was not significantly different between the two groups (*P* > 0.05). All adverse events were resolved at the end of follow-up period. None of the other participants reported any adverse reactions.

## 4. Discussion

In our study, we evaluated and compared the efficacy of fosinopril or amlodipine monotherapy in children and adolescents with primary hypertension for the first time. We found significant reductions in SBP and DBP after 4 weeks of treatment with fosinopril or amlodipine, with BP control rates of 61.5% and 59.5%, respectively. Furthermore, the fosinopril group exhibited higher, at least trend, reductions in SBP and DBP, and DBP control rates among subgroups of females, patients with central obesity, IR, and hypo-HDL-cholesterolemia, in comparison to the amlodipine group.

Fosinopril has a rapid onset of action and is the first-line antihypertensive drug in clinical practice. Previous studies have shown that fosinopril can lower BP levels and is well tolerated in both adults and children (22, 23). Li et al. found that fosinopril significantly reduced SBP and DBP in hypertensive children, with BP control rate of 47% at 4 weeks of medium-dose (0.1–0.3 mg/kg) administration (23). In our study, the BP control rate of fosinopril were 61.5% after 4 weeks of treatment with median dosage of 0.27 mg/kg, higher than that in their study. Of note, 17.2% patients had high-normal BP with an associated clinical condition in their study, while our subjects were all hypertensive children, which may be speculated as the possible reason for the difference in BP control rate. Additionally, their study did not distinguish BP control rate in different groups of primary hypertension and renal etiology for hypertension, whereas our study is more reflective of the efficacy of fosinopril in pediatric primary hypertension.

Amlodipine is known to induce peripheral vasodilation and improve hypertension-related endothelial dysfunction (24, 25). A large-scale epidemiological survey in China showed that CCB were the most commonly used antihypertensive drugs in adults with hypertension, with BP control rate of 45.8% (26). Amlodipine is acknowledged as a first-line antihypertensive drug, but there are few reports on its efficacy in treating pediatric primary hypertension. The systematic review showed that amlodipine achieves significant decrease in both SBP and DBP in children with different disease states, but limited evidence suggested that the BP control rate is only 30%–50% (7). Flynn et al. found that treatment with amlodipine at a dose of 2.5 mg or 5 mg daily for 8 weeks significantly reduced SBP in hypertensive children, with BP, SBP and DBP control rates in children with primary hypertension of 8.3%, 33.3%, and 45%, respectively (19), while 59.5%, 63.3%, and 55.6% in our 4-week study. Our initial dosage was 5 mg daily and titrated gradually according to BP levels, which may be the reason for the higher control rates in our study.

To our knowledge, this is the first clinical report comparing the efficacy of fosinopril and amlodipine in the treatment of children with primary hypertension. PavlovićK et al. found no difference in BP control rates between fosinopril or amlodipine monotherapy for 3 months in patients older than 60 years with isolated systolic hypertension (76.6% vs. 79.9%) (27). Similar to their results, we found no difference in BP control rates between the two groups, but a 10% difference in the degree of SBP and DBP reductions, although this difference was no longer significant after adjustment. However, given that the small sample size may have limited the power to determine significant between-group differences, we considered the difference to be trending toward significance and may evolve as the sample size increased. Taken together, we believed that there should be some differences in certain populations, and in this regard, we conducted subgroup analyses based on demographic characteristics. Considering that most of our subjects were adolescents, we did not perform age-stratified subgroup analysis. The results showed that BP reductions were significantly greater with fosinopril in female and central obesity subgroups. Considering the mechanism of both drugs, we speculate that it may be the blockade of Renin-angiotensin System (RAS) that mediates this difference, at least partially.

RAS activation is an important mechanism for the development of hypertension in children. ACE, the main component of RAS, converts angiotensin I into angiotensin II (Ang II), which binds to its receptor to cause vasoconstriction, and release of aldosterone, resulting in a rise in BP. RAS is known to have sex differences, and endogenous sex hormones have been shown to interact with RAS, which can be upregulated by androgens and antagonized by estrogens (28, 29). However, there is limited data on the influence of sex on efficacy of antihypertensive drugs. A meta-analysis of clinical trials with sex-specific outcomes showed a slight increase in cardiovascular benefit of ACEI in male compared with female (30). However, that the majority of women in these trials were postmenopausal, and anti-RAS therapy may vary based on the reproductive status of female. Limited evidence suggests that female may exhibit a greater response to anti-RAS therapy than male in younger subjects. Miller et al. found more effective angiotensin receptor blockade in response to irbesartan administration in female than in male in a population of normotensive young participants (mean age in female, 28 ± 2 years) (31). In a trial of efficacy in young healthy volunteers, it was observed that the effectiveness of enalapril increased in females as plasma concentrations increased over time, with lower SBP levels and ACE activity at the time of maximal BP-lowering effects than in male (32). Thus, although we are not yet able to explain this sex-induced difference in efficacy, it cannot be denied that understanding this difference may lead to better treatment options for children.

It is estimated that at least 75% of the incidence of hypertension in children is directly related to obesity. Obese patients are prone to develop hypertension, which is mainly attributed to the excessive activation of RAAS. In obesity, adipose tissue produces large amounts of AngII, and plasma RAS levels are elevated, especially those with central obesity (33). In addition, obese children are often accompanied by metabolic abnormalities such as IR, elevated triglycerides, and decreased HDL-C (34–36). Studies have shown that IR and its concomitant hyperinsulinemia tend to enhance sympathetic tone, RAS activity, and sodium and water reabsorption at the renal tubular level, leading to a progressive increase in vascular stiffness that further raises BP (37). And hyperlipidemia contributes to the activation of intrarenal RAS but not circulating RAS, which further aggravates water and sodium reabsorption and renal injury (38). Our subsequent subgroup analysis also demonstrated a greater, at least trend, reduction in BP with fosinopril in patients with central obesity, IR and hypo-HDL-cholesterolemia. However, there have been no clinical trials to date comparing the efficacy of ACEI with CCBs in lean and obese patients, especially in pediatric primary hypertension. Our results may therefore have implications for the selection of antihypertensive agents for obesity-related hypertensive children and adolescents.

There are several limitations in this study, including that this is a hospital-based, single-center observational study that cannot completely avoid selection bias, and further multicenter, randomized large-scale trials are necessary. The small sample size also limited the power of subgroup analyses, and future studies with large sample sizes and predefined subgroup analyses are needed to confirm or refute our findings. The antihypertensive efficacy may be affected by individual gene polymorphisms, such as *ACE1* and *CYP3A5*, and study on this aspect is currently ongoing. However, this study may help guide pediatricians to choose antihypertensive agents according to the different clinical characteristics of hypertensive children and was expected to improve hypertension treatment.

## 5. Conclusion

Fosinopril and amlodipine monotherapy were both effective in pediatric primary hypertension during a short-term follow-up. Fosinopril may be particularly effective in reducing BP in hypertensive patients of females, central obesity, insulin resistance, and hypo-HDL-cholesterolemia. These findings indicate that the optimization of antihypertensive medication selection in conjunction with the individualized characteristics of children with hypertension may improve the efficacy of antihypertensive treatment.

## Data Availability

The original contributions presented in the study are included in the article/**Supplementary Material**, further inquiries can be directed to the corresponding author.
