# Supplementary material for: Efficacy of fosinopril and amlodipine in pediatric primary hypertension: a single-center observational study
Source: Front Pediatr. 2023 Oct 27;11:1247192. doi: 10.3389/fped.2023.1247192 (PMC10641687; doi:10.3389/fped.2023.1247192)
Supplement: Supplementary file 1 [file Datasheet1.docx]

Supplementary Material

**Efficacy of fosinopril and amlodipine in pediatric primary hypertension: a single-center observational study**

Hui Wang^1^, Lin Shi^1,2*^, Yao Lin^2^, Yuting Wang^1^, Wenquan Niu^3^, Yaqi Li^2^

*** Correspondence:** Lin Shi, email: [shilin9789@126.com](mailto:shilin9789@126.com])

**Supplementary Table 1** Subgroup analyses for BP reductions and control rates after treatment in the fosinopril or amlodipine groups

| **Subgroup** | **Characteristic** | **Fosinopril** | **Amlodipine** | ***P*-value** | **adjusted**  ***P*-value** |
| --- | --- | --- | --- | --- | --- |
| **Gender** |  |  |  |  |  |
| **Male (n=139)** | Age (years) | 13.0 (2.0) | 13.0 (2.0) | 0.783 |  |
|  | Baseline BMI (kg/m^2^) | 28.50±4.51 | 28.44±5.20 | 0.948 |  |
|  | Central obesity (n, %) | 60 (81.1) | 48 (73.8) | 0.307 |  |
|  | Baseline SBP (mmHg) | 144.5 (14.5) | 144.0 (12.0) | 0.411 |  |
|  | Baseline DBP (mmHg) | 80.0 (20.0) | 80.0 (13.5) | 0.384 |  |
| **Female (n=36)** | Age (years) | 13.0 (3.0) | 14.0 (4.8) | 0.962 |  |
|  | Baseline BMI (kg/m^2^) | 26.76±4.70 | 29.62±5.03 | 0.093 |  |
|  | Central obesity (n, %) | 15 (68.2) | 13 (92.9) | 0.115 |  |
|  | Baseline SBP (mmHg) | 143.5 (11.0) | 138.0 (12.5) | 0.077 |  |
|  | Baseline DBP (mmHg) | 89.0 (10.5) | 83.5 (10.5) | 0.160 |  |
| **Central obesity** |  |  |  |  |  |
| **Yes (n=136)** | Age (years) | 13.0 (2.0) | 13.0 (3.0) | 0.413 |  |
|  | Male (n, %) | 60 (80.0) | 48 (78.7) | 0.851 |  |
|  | Baseline BMI (kg/m^2^) | 29.73±3.61 | 30.29±4.66 | 0.449 |  |
|  | Baseline SBP (mmHg) | 146.0 (14.0) | 144.0 (13.0) | 0.144 |  |
|  | Baseline DBP (mmHg) | 80.0 (18.0) | 80.0 (13.5) | 0.168 |  |
| **No (n=39)** | Age (years) | 13.0 (2.5) | 14.0 (2.0) | 0.148 |  |
|  | Male (n, %) | 14 (66.7) | 17 (94.4) | 0.049 |  |
|  | Baseline BMI (kg/m^2^) | 22.26±2.48 | 23.11±1.83 | 0.239 |  |
|  | Baseline SBP (mmHg) | 142.0 (14.0) | 142.0 (14.3) | 0.989 |  |
|  | Baseline DBP (mmHg) | 90.0 (14.0) | 80.0 (13.0) | 0.443 |  |
| **Insulin resistance** |  |  |  |  |  |
| **Yes (n=127)** | Age (years) | 13.0 (2.0) | 13.0 (2.0) | 0.588 |  |
|  | Male (n, %) | 54 (76.1) | 46 (82.1) | 0.405 |  |
|  | Baseline BMI (kg/m^2^) | 29.25±4.10 | 30.14±4.94 | 0.283 |  |
|  | Central obesity (n, %) | 63 (88.7) | 50 (89.3) | 0.921 |  |
|  | Baseline SBP (mmHg) | 144.0 (12.0) | 144.0 (13.0) | 0.315 |  |
|  | Baseline DBP (mmHg) | 82.0 (18.0) | 80.0 (14.0) | 0.185 |  |
|  | d-SBP (mmHg) | 21.6±11.31 | 17.5±13.15 | 0.060 | 0.100 |
|  | d-DBP (mmHg) | 9.2±12.23 | 4.7±10.88 | 0.032 | 0.080 |
|  | BP control rate (%) | 46 (64.8) | 31 (55.4) | 0.280 | 0.350 |
|  | SBP control rate (%) | 47 (66.2) | 33 (58.9) | 0.400 | 0.400 |
|  | DBP control rate (%)* | 39 (83.0) | 17 (54.8) | 0.007 | 0.035 |
| **No (n=48)** | Age (years) | 14.0 (3.0) | 14.0 (3.0) | 0.362 |  |
|  | Male (n, %) | 20 (80.0) | 19 (82.6) | 1.000 |  |
|  | Baseline BMI (kg/m^2^) | 24.83±4.42 | 25.04±3.76 | 0.862 |  |
|  | Central obesity (n, %) | 12 (48.0) | 11 (47.8) | 0.990 |  |
|  | Baseline SBP (mmHg) | 147.0 (14.5) | 142.0 (12.0) | 0.373 |  |
|  | Baseline DBP (mmHg) | 88.0 (22.0) | 80.0 (9.0) | 0.468 |  |
|  | d-SBP | 19.5±9.74 | 19.4±9.22 | 0.950 | 0.950 |
|  | d-DBP | 8.6±12.22 | 9.4±11.47 | 0.837 | 0.950 |
|  | BP control rate | 13 (52.0) | 16 (69.6) | 0.214 | 0.535 |
|  | SBP control rate | 14 (56.0) | 17 (73.9) | 0.195 | 0.535 |
|  | DBP control rate* | 10 (62.5) | 8 (57.1) | 0.765 | 0.950 |
| **Hypertriglyceridemia** |  |  |  |  |  |
| **Yes (n=64)** | Age (years) | 13.0 (2.0) | 13.0 (3.5) | 0.520 |  |
|  | Male (n, %) | 23 (74.2) | 27 (81.8) | 0.461 |  |
|  | Baseline BMI (kg/m^2^) | 29.24±2.53 | 29.55±4.70 | 0.744 |  |
|  | Central obesity (n, %) | 30 (96.8) | 30 (90.9) | 0.651 |  |
|  | Baseline SBP (mmHg) | 147.0 (17.0) | 144.0 (14.5) | 0.234 |  |
|  | Baseline DBP (mmHg) | 80.0 (20.0) | 80.0 (16.5) | 0.887 |  |
|  | d-SBP | 22.7±11.29 | 20.0±11.58 | 0.342 | 0.798 |
|  | d-DBP | 8.9±12.59 | 7.9±10.96 | 0.745 | 0.846 |
|  | BP control rate | 19 (61.3) | 21 (63.6) | 0.846 | 0.846 |
|  | SBP control rate | 19 (61.3) | 23 (69.7) | 0.479 | 0.798 |
|  | DBP control rate* | 15 (75.0) | 12 (60.0) | 0.311 | 0.798 |
| **No (n=111)** | Age (years) | 13.0 (2.5) | 14.0 (3.0) | 0.418 |  |
|  | Male (n, %) | 51 (78.5) | 38 (82.6) | 0.589 |  |
|  | Baseline BMI (kg/m^2^) | 27.55±5.23 | 28.00±5.43 | 0.659 |  |
|  | Central obesity (n, %) | 45 (69.2) | 31 (67.4) | 0.837 |  |
|  | Baseline SBP (mmHg) | 144.0 (12.0) | 143.0 (12.0) | 0.434 |  |
|  | Baseline DBP (mmHg) | 86.0 (15.5) | 80.0 (9.5) | 0.057 |  |
|  | d-SBP | 20.3±10.72 | 16.6±12.40 | 0.100 | 0.167 |
|  | d-DBP | 9.2±12.06 | 4.7±11.28 | 0.053 | 0.133 |
|  | BP control rate | 40 (61.5) | 26 (56.5) | 0.596 | 0.596 |
|  | SBP control rate | 42 (64.6) | 27 (58.7) | 0.526 | 0.596 |
|  | DBP control rate* | 34 (79.1) | 13 (52.0) | 0.020 | 0.100 |
| **Hypo-HDL-cholesterolemia** |  |  |  |  |  |
| **Yes (n=83)** | Age (years) | 13.0 (2.0) | 13.0 (2.8) | 0.899 |  |
|  | Male (n, %) | 38 (80.9) | 30 (83.3) | 0.771 |  |
|  | Baseline BMI (kg/m^2^) | 28.83±4.41 | 30.81±4.59 | 0.051 |  |
|  | Central obesity (n, %) | 42 (89.4) | 34 (94.4) | 0.669 |  |
|  | Baseline SBP (mmHg) | 146.0 (14.0) | 144.0 (11.5) | 0.355 |  |
|  | Baseline DBP (mmHg) | 82.0 (22.0) | 78.0 (15.5) | 0.104 |  |
|  | d-SBP | 23.1±9.58 | 17.2±12.86 | 0.018 | 0.045 |
|  | d-DBP | 10.3±12.19 | 3.9±11.84 | 0.018 | 0.045 |
|  | BP control rate | 33 (70.2) | 22 (61.1) | 0.385 | 0.481 |
|  | SBP control rate | 33 (70.2) | 23 (63.9) | 0.542 | 0.542 |
|  | DBP control rate* | 26 (86.7) | 9 (56.3) | 0.052 | 0.087 |
| **No (n=92)** | Age (years) | 13.0 (2.0) | 13.0 (3.0) | 0.759 |  |
|  | Male (n, %) | 36 (73.5) | 35 (81.4) | 0.366 |  |
|  | Baseline BMI (kg/m^2^) | 27.40±4.70 | 26.84±4.96 | 0.584 |  |
|  | Central obesity (n, %) | 33 (67.3) | 27 (62.8) | 0.647 |  |
|  | Baseline SBP (mmHg) | 144.0 (14.0) | 142.0 (13.0) | 0.357 |  |
|  | Baseline DBP (mmHg) | 84.0 (12.5) | 80.0 (10.0) | 0.532 |  |
|  | d-SBP | 19.1±11.81 | 18.7±11.55 | 0.890 | 0.996 |
|  | d-DBP | 7.9±12.16 | 7.9±10.39 | 0.996 | 0.996 |
|  | BP control rate | 26 (53.1) | 25 (58.1) | 0.625 | 0.996 |
|  | SBP control rate | 28 (57.1) | 27 (62.8) | 0.581 | 0.996 |
|  | DBP control rate* | 23 (69.7) | 16 (55.2) | 0.237 | 0.996 |

BP: blood pressure. BMI: body mass index. d-SBP: decrease of systolic blood pressure from baseline to week 4. d-DBP: decrease of diastolic blood pressure from baseline to week 4. * indicates DBP control rate in systolic and diastolic hypertension. HDL: high-density lipoprotein. The continuous variables were presented as mean ± standard deviation.
